# Contralateral C7 nerve transfer in the treatment of central hemiplegia after stroke under general anesthesia: A case report

**DOI:** 10.1002/ibra.12064

**Published:** 2022-08-24

**Authors:** Qiu‐Ying Zhang, Yi Guo, Yi‐Nan Zhang

**Affiliations:** ^1^ Department of Anesthesiology Affiliated Hospital of Zunyi Medical University Zunyi Guizhou China; ^2^ Department of Hepatological Surgery Affiliated Hospital of Zunyi Medical University Zunyi Guizhou China

**Keywords:** C7 nerve transfer, cerebral hemiplegia, cerebral stroke, general anesthesia

## Abstract

Similar reports in the past pay less attention to the anesthetic management of these patients. We reported a 46‐year‐old man who suffered from hypertensive cerebral apoplexy 5 months ago and accepted C7 nerve transfer to improve the central spastic paralysis in the right upper limb. After careful evaluation and anesthesia management before anesthesia, the operation was successfully completed under general anesthesia. The patient was cured and discharged without complications. The anesthesia management of C7 nerve transfer should choose appropriate operation opportunities for patients according to the type of stroke, improve the preoperative preparation, and form a multidisciplinary diagnosis and treatment.

## Graphical abstract

Cervical 7 nerve transposition is a new operation, and due to the particularity of patient complications, we should specify a suitable anesthesia plan for patients, hence we came to the following conclusions through the anesthesia management of this patient. We had to form a multidisciplinary diagnosis and treatment to determine the operation time and formulate the operation plan, then reduce the perioperative complications of patients, and eventually, achieve the purpose of enhanced recovery after surgery.

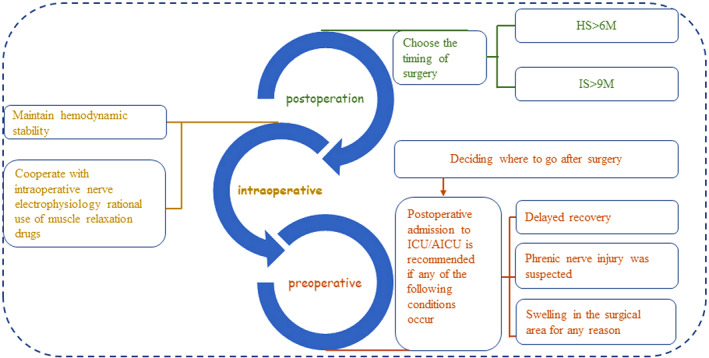

## 1 INTRODUCTION

Stroke remains a major cause of disability in worldwide. Hemiplegia is one of the most common adverse outcomes, which brings a great burden to society and family. The team of Gu Yudong^1^ used C7 nerve transfer to treat central upper limb paralysis for the first time in 2018. C7 nerve transfer surgery even though improves the function of upper limbs can also affect muscle properties of lower limbs of spastic hemiplegia.^2^ The safety of operation, the combined medical history, and the particularity of operation have been receiving increasing attention because of the significant influence on prognosis. We reported the case of a patient who underwent C7 nerve transplantation under general anesthesia to improve the limb movement disorder after stroke.

## 2 CASE PRESENTATION

**Figure 1:**
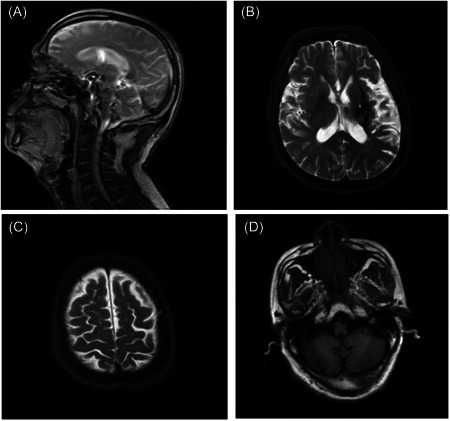
MRI images at admission. MRI showed encephalatrophy, multiple old bleeding, and softening lesions occurred in the left basal ganglia region, coronaradiata and brainstem, bilateral white matter hemorrhage, and demyelinating lesions. MRI, magnetic resonance imaging.

A 46‐year‐old male patient, 168 cm, 65 kg, American Society of Anesthesiologists grade of physical status III, suffered from hypertensive cerebral apoplexy 5 months ago, and his right limb became hemiplegic after the operation. To improve the motor function of the right limb, the patient was admitted to the hospital on April 18, 2021. He suffered from hypertension 2 years ago, and after the operation 5 months ago, his blood pressure was controlled by regular use of spironolactone, enalapril, metoprolol, nifedipine, and atorvastatin. Relevant examinations were completed after he was admitted to the hospital. There was no obvious abnormality in the blood routine and biochemical examination. The auxiliary examination includes a normal chest radiograph, electrocardiogram showing T‐wave changes, cranial magnetic resonance imaging (MAGENTON Avanto Dot, SIEMENS) showing brain atrophy, multiple old hemorrhages and softening foci in the left basal ganglia, radiation crown and brain stem, and bilateral white matter hemorrhage and demyelination (Figure 1). The patient underwent C7 nerve transfer under general anesthesia and endotracheal intubation on April 23, 2021.

## 3 ANESTHESIA AND SURGICAL PROCESS

**Figure 2:**
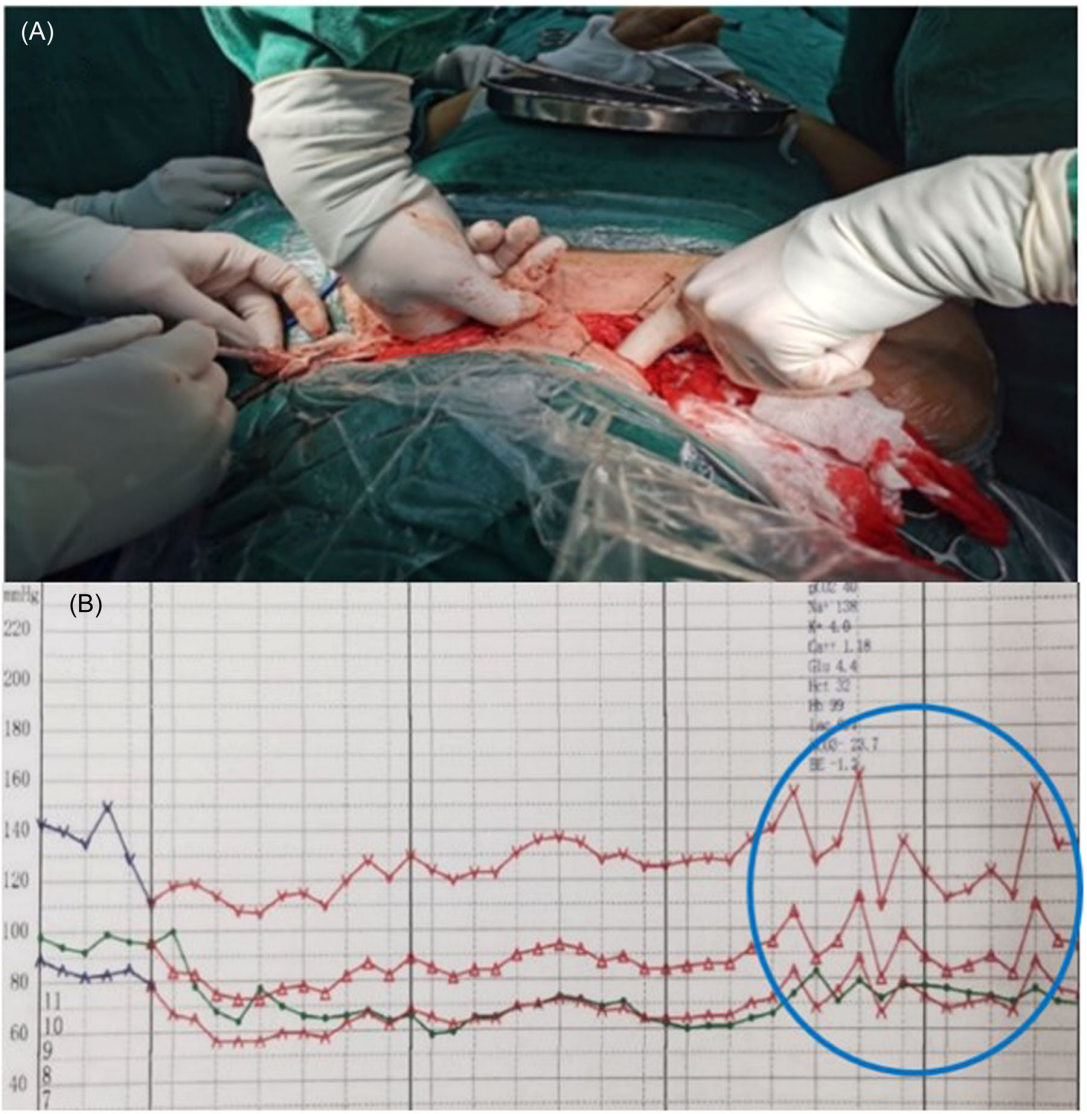
Changes in vital signs in establishing nerve tunnel. Established tunnel before nerve anastomosis. (B) The circle was marked with the changes in vital signs when the tunnel was built. IBP fluctuated at 110−162/65−91 mmHg. IBP, invasive blood pressure. [Color figure can be viewed at wileyonlinelibrary.com]

His basic vital signs were recorded as: noninvasive blood pressure 140/82 mmHg, heart rate (HR) 93 bpm, pulse oxygen saturation (SpO_2_) 98%, respiratory rates 20 bpm. Anesthesia was induced by intravenous injection of midazolam 2 mg, sufentanil 25 mg, etomidate 16 mg, and rocuronium 50 mg. The endotracheal tube was successfully placed and invasive mechanical ventilation was performed immediately. The volume‐controlled ventilation mode was adopted, and the invasive blood pressure (IBP) was monitored by an arterial puncture. The anesthesia was maintained as a combination of propofol 4−6 mg/kg/h, remifentanil 0.2−0.3 µg/kg/min, dexmedetomidine 0.5 mg/kg/h, and sevoflurane 0.5−1.0 minimal alveolar concentration. The surgeon exposed bilateral brachial plexus nerves after the anesthetic kicks in. The C7 nerve root from the unaffected arm is dissected distally until the level of the brachial plexus divisions. The C7 nerve root on the affected side is then transected at the exit from the neural foramina. The donor C7 root is then tunneled via the prevertebral space (with a sural nerve graft for tension‐free suture), to the contralateral side and coapted to the recipient distal end of the C7 root on the affected side. Muscle relaxants were not used during the operation. The anesthesia process was relatively smooth, but before the nerve anastomosis, IBP fluctuated at 110−62/65−91 mmHg (Figure 2B) when the tunnel was established (Figure 2A), and it lasted an hour. There was no obvious fluctuation in HR, and sufentanil 10 μg was added. The operation lasted for 8 h and 30 min. The total amount of propofol was 2000 mg, remifentanil 4 mg, dexmedetomidine 0.2 mg, and sevoflurane 100 ml. One hour after the operation, the patient regained his spontaneous breathing, but his consciousness was not well enough. Considering the possibility of phrenic nerve injury, edema in the operating area may compress the airway. After the operation, the patient was sent to the intensive care unit (ICU) and inserted with an endotracheal tube. We followed up with the patients on the first day after the operation. The tracheal tube had been removed, the patient was conscious, the vital signs were stable, the operating area was slightly painful, and there was no nausea and vomiting. The patient was discharged on the 10th day postoperation. At 1 year follow‐up, the degree of spasticity was improved compared with pre‐operation.

## 4 DISCUSSION

We report a patient with hemiplegia of the right limb caused by hypertension. To improve the right upper central spastic paralysis, the patient chose the contralateral C7 nerve transfer. The anesthesia for this type of surgery has rarely been reported in the past. We formed a multidisciplinary diagnosis and treatment (MDT) and developed appropriate anesthesia and surgical plan for the patient to promote postoperative recovery and discharge. Post‐stroke hemiplegia brings inconvenience to patients’ lives, and they cannot even take care of themselves. C7 nerve transplantation can greatly improve the motor function and coordination of the upper limbs of stroke patients with hemiplegia,^1^ which brings great benefits to stroke patients with hemiplegia. This is not only beneficial to family but also has huge social benefits. However, in different types of patients with stroke history, intraoperative complications may occur.

## 5 PREOPERATIVE PREPARATION

The patient's type and history of stroke and optimal timing of surgery need to be known before surgery, in addition to the relevant examination. The types of strokes include hemorrhagic stroke (HS) and ischemic stroke (IS). No matter what kind of stroke, the slight fluctuation of cerebral perfusion pressure can aggravate brain injury because the ability of cerebral autoregulation (CA) is impaired after stroke. Previous studies have shown that the impairment of CA after stroke can last for 1−6 months.^3^, ^4^

## 6 PREOPERATIVE PREPARATION FOR HS PATIENTS

At present, there is no relevant report on the timing of surgery for nerve transfer surgery in patients with HS. Stroke patients can improve on some limb dysfunction through functional exercises. The surgeon believes that if the patient can no longer improve limb function after stroke, the limb dysfunction returns to the plateau phase,^5^ and the nerve transposition surgery can be selected. Although there is no uniform standard, the blood pressure of such patients should be well controlled because the CA and main artery elasticity have decreased, and high or low blood pressure may cause cerebrovascular accidents; especially when the anterior nerve tunnel is established, the pressure on the carotid and the jugular vein causes blood pressure fluctuation, which, in turn, increases the risk for patients with insufficient CA. According to the 2018 Chinese Guidelines for Prevention and Treatment of Hypertension, the goal for lowering BP should be less than 140/90 mmHg^6^ for stable stroke patients.

## 7 PREOPERATIVE PREPARATION FOR IS PATIENTS

It should be based on the causes of stroke, location of stenosis vessels, and degree of vascular stenosis to judge the risk of thrombosis recurrence. A large sample observational study found that patients with a history of IS were associated with adverse outcomes following surgery, in particular, if the time between IS and surgery was less than 9 months. After 9 months, the associated risk appeared stable yet still increased compared with patients with no IS.^7^ So, patients with a history of IS who need to undergoing surgery should be more than 9 months apart from stroke. IS patients have to take anticoagulants for a long time before the operation. Therefore, we also need to consider whether to stop anticoagulants before surgery at first and whether to carry out bridging therapy. Because of the complicated condition, the patient can apply for multidisciplinary consultation, such as related surgery, neurology, neurosurgery, anesthesiology, vascular surgery, and so forth, to determine the operation time, formulate the operation plan, evaluate the risk of thromboembolism during the perioperative period, and guide the medication before and during operation.

## 8 ANESTHESIA MANAGEMENT

Anesthesia management includes the choice of anesthetic and the management of intraoperative circulation. Many anesthetics have protective effects on the brain,^8^, ^9^, ^10^ but there are few reports about the effects of anesthetics on patients with a history of stroke. The operation requires electrophysiology to find the C7 nerve accurately. The use of muscle relaxants in neuroelectrophysiological surgery is still inconclusive. Generally speaking, if combined with neuroelectrophysiology, muscle relaxants should be avoided. Normally, this type of surgery is performed with a microscope because of the proximity of large blood vessels. Without the use of muscle relaxants, the possible intraoperative body movement can cause greater harm. To avoid this situation, it is usually necessary to maintain a deep depth of anesthesia. If a muscle relaxant is used normally, it is necessary to communicate closely with the surgeon to stop using it at the appropriate time to avoid the effect on neuroelectrophysiology. Some studies have suggested that the partial neuromuscular blockade, that is, partial neuromuscular junction blocking and maintaining T1/Tc 20%−40% during facial nerve microvascular decompression can effectively reduce the limb movement response during operation and improve perioperative safety.^11^ It may be used to transfer the seventh cervical nerve, but its effectiveness and safety still need to be further studied. In this case, a muscle relaxant was not used in the anesthesia maintenance, and the patient did not have any limb movement reaction, but the patient woke up 1 h after the operation. We may keep deep anesthesia during the operation, which is not conducive to the rapid recovery of the patient. Previous stroke history is a risk factor for perioperative stroke. To reduce the risk of perioperative stroke, blood pressure management should be the focus of perioperative management. Intraoperative blood pressure should be stable, or systolic blood pressure should be controlled at no less than 20% of the basic value to ensure sufficient cerebral perfusion pressure.^12^

## 9 POSTOPERATIVE MANAGEMENT

There are few reports about the postoperative treatment of these patients. Postoperative management should first consider where patients will go after surgery, and whether the patients will return to the ward or ICU or Anesthesiology Intensive Treatment Unit (AICU). The patient was admitted to the ICU due to postoperative unconsciousness. At the same time, we have also considered the possibility of phrenic nerve injury during an operation.

As the phrenic nerve dominates the diaphragm, it plays an important role in regulating respiratory function. If phrenic nerve injury is suspected, it may be necessary to retain the endotracheal tube. In addition, the surgical area may be swollen after an operation. If the endotracheal tube is pulled out prematurely, there is a risk of upper respiratory obstruction. Therefore, it is necessary to comprehensively consider patient factors, surgical factors, and anesthesia factors to determine the postoperative destination of patients.

## 10 SUMMARY

In the anesthesia management of this surgical patient, we consulted the relevant treatment and worked closely with the surgeon to develop the best anesthesia plan for the patient so that the patient could be safe in the perioperative period. Patients with a history of stroke should consult with relevant departments before C7 nerve transposition operation, form an MDT, determine the operation time, formulate the operation plan, the perioperative complications of patients, and achieve the goal of enhanced recovery after surgery.

## AUTHOR CONTRIBUTIONS

Qiu‐Ying Zhang is responsible for the data collection, data curation, writing, and revision. Yi Guo is responsible for the conceptual framework and graphic production. Yi‐Nan Zhang is responsible for supervising and revising the manuscript.

## CONFLICT OF INTEREST

The authors declare no conflict of interest.

## ETHICS STATEMENT

All experimental procedures were approved by the Ethical Committee of Affiliated Hospital of Zunyi Medical University (No: KLL‐2022‐573). The patient has signed the informed consent.

## Data Availability

The data generated and analyzed in the present study can be obtained from the corresponding author.
